# Nostril-Anchored Geometric ROI Projection for Contactless Forehead Skin Temperature Estimation Using Low-Resolution Thermal Imaging

**DOI:** 10.3390/s26165147

**Published:** 2026-08-14

**Authors:** Riska Analia, Anne Forster, Sheng-Quan Xie, Zhiqiang Zhang

**Affiliations:** 1 School of Electronic and Electrical Engineering, University of Leeds, Leeds LS2 9JT, UK; elran@leeds.ac.uk (R.A.); s.q.xie@leeds.ac.uk (S.-Q.X.); 2 Department of Electrical Engineering, Politeknik Negeri Batam, Batam 29461, Indonesia; 3 Academic Unit for Ageing and Stroke Research, Leeds Institute of Health Sciences, University of Leeds, Leeds LS2 9LN, UK; a.forster@leeds.ac.uk

**Keywords:** contactless thermal sensing, geometric ROI projection, forehead skin temperature, facial anthropometry, low-resolution thermal camera, edge computing, real-time deployment

## Abstract

(1) Background: Reliable forehead region-of-interest (ROI) localization remains challenging in low-resolution thermal imaging because limited spatial detail increases localization uncertainty. This study develops a lightweight nostril-anchored geometric ROI projection method for contactless forehead skin temperature estimation. (2) Methods: The forehead ROI was projected from the detected nostril bounding box using anthropometry-guided proportional geometry, followed by 95th-percentile ($P_{95}$) spatial aggregation and exponential moving average (EMA) stabilization. Direct localization was evaluated against fixed-crop and MediaPipe Pose-based baselines using 120 annotated pilot frames across 0.5–1.5 m, followed by validation on 150 additional annotated frames from ten formal participants. A medical non-contact infrared forehead thermometer targeting the same central forehead region was used as a practical, rather than traceably calibrated, comparator. (3) Results: The proposed method outperformed the two localization baselines in the pilot evaluation. Formal-cohort validation achieved an overall IoU of 0.714, FIR of 0.870, and NCE of 0.091, with a 0.7% spatial-failure rate. Across 90 temperature measurements, the method achieved an MAE of 0.514 °C, RMSE of 0.614 °C, and mean bias of −0.187 °C, with 95% Bland–Altman limits of agreement from −1.340 °C to 0.966 °C. Real-time implementation achieved 24.12±1.43 FPS on a Jetson Orin Nano. (4) Conclusions: The proposed method provides a computationally efficient forehead ROI localization approach for low-resolution thermal imaging. Localization was strongest at 0.5 m and lower at farther distances; therefore, its scale adaptation should not be interpreted as distance-invariant performance. The temperature results represent agreement with the practical comparator rather than absolute clinical accuracy.

## 1. Introduction

Contactless physiological sensing is increasingly relevant to smart environments and embedded monitoring systems. Thermal imaging is a promising sensing modality because it captures surface temperature distributions without requiring wearable sensors or direct physical contact [1,2,3]. Compared with conventional RGB cameras, thermal imaging can provide improved visual privacy while preserving physiologically meaningful surface-temperature information. However, practical deployment remains challenging because thermal cameras generally have lower spatial resolution, reduced facial detail, and greater sensitivity to environmental conditions. These limitations make stable facial region-of-interest (ROI) localization particularly important when low-resolution thermal cameras are used for contactless monitoring.

Forehead skin temperature is commonly used as a practical non-invasive surface measurement [4]. Although forehead skin temperature does not directly represent core body temperature, it provides an accessible facial measurement site for contactless temperature estimation under controlled conditions [5]. In this study, the forehead was selected because it provides a relatively large and accessible facial surface, which is advantageous for low-resolution thermal imaging where smaller regions, such as the inner canthus, may contain too few pixels for stable automatic localization [6]. However, the reliability of the extracted forehead temperature depends strongly on stable and anatomically meaningful ROI localization because measured facial surface temperature varies with the selected region and its spatial consistency [7]. Accordingly, the temperature values reported in this study are interpreted as localized forehead skin temperature estimates rather than as measurements of core body temperature or clinical status.

Existing forehead ROI localization approaches provide useful solutions under suitable imaging conditions, but they differ in anatomical anchoring, sensing requirements, and suitability for embedded deployment. Image-processing-based forehead segmentation can directly extract the forehead region from thermal images and may operate in real time on embedded hardware [8]. However, such methods may depend on stable head shape segmentation, eye or glasses detection, and assumptions about facial geometry. Landmark-based and key-region localization methods can improve anatomical precision by using facial landmarks, eye/canthus regions, or multimodal facial cues, but their performance depends on the visibility and stability of facial structures and, in some cases, additional sensing or calibration [9,10,11]. Automatic facial ROI detection methods can localize predefined facial regions, including the forehead, but they generally require task-specific annotated thermal datasets and reliable face-region detection [12]. Therefore, these approaches commonly depend on direct forehead or face localization, stable facial landmarks, multimodal sensing, or task-specific thermal annotations. Their reliability may decrease in low-resolution thermal images, where hair interference, boundary leakage, distance-related scale changes, pixel-level noise, and head pose variation can affect ROI consistency [13,14,15].

To address this localization challenge, this paper proposes a nostril-anchored geometric ROI projection method for contactless forehead skin temperature estimation. The proposed method uses the detected nostril region as a thermal and anatomical anchor from which a forehead ROI whose dimensions and position adapt proportionally to the apparent nostril scale is projected using anthropometry-guided geometric relationships. The nostril region is thermally distinctive because respiratory airflow produces localized temperature variation around the nasal area, making it detectable in facial thermal imagery [16,17]. It also provides a lower facial reference point that is spatially related to the forehead through craniofacial proportions [18]. Rather than detecting the forehead directly or estimating dense facial landmarks, the proposed method uses the detected nostril bounding box as an image-domain geometric anchor to determine the position and scale of the projected forehead ROI.

The objective of this work is to develop and directly evaluate a lightweight nostril-anchored method for forehead ROI localization in low-resolution thermal images. Direct spatial localization is first assessed against manually annotated forehead regions and compared with fixed-crop and landmark-based baselines during the pilot evaluation. Generalization of the selected method is subsequently evaluated across all ten participants in the formal cohort under the three frontal camera-to-subject distances. Forehead skin temperature estimation is then evaluated as a downstream application under variations in camera distance, natural non-frontal head orientation, and posture, together with real-time embedded implementation. A medical non-contact infrared forehead thermometer targeting approximately the same central forehead region was used as a practical comparator. Because the handheld device and the thermal-camera method differ in spatial sampling and measurement principle, the comparator was not treated as a gold-standard or traceably calibrated reference. Accordingly, the temperature-related metrics are interpreted as agreement between the two forehead temperature measurement approaches under the evaluated conditions rather than as absolute clinical or metrological accuracy. The main contributions of this study are the following: (i) a nostril-anchored geometric method for forehead ROI projection in low-resolution thermal images; (ii) direct annotation-based spatial evaluation using intersection over union (IoU), forehead inclusion ratio (FIR), and normalized center error (NCE), including a pilot comparison with fixed-crop and landmark-based baselines and subsequent generalization assessment across the ten-participant formal cohort; (iii) evaluation of the nostril anchor and projected forehead ROI across camera-to-subject distances; and (iv) application-level evaluation of forehead skin temperature estimation under variations in camera distance, natural head orientation, and posture, together with real-time implementation on a Jetson Orin Nano.

## 2. Related Work

Reliable forehead skin temperature estimation in thermal imaging depends not only on the thermal sensor itself but also on consistent and anatomically meaningful ROI localization. Although infrared thermography (IRT) enables contactless skin temperature measurement [19,20], the measured temperature can vary substantially with ROI definition, camera-to-subject distance, sensor characteristics, environmental conditions, and measurement protocol [21,22]. Even small localization errors may lead to noticeable differences in the extracted forehead temperature, particularly when low-resolution thermal cameras provide only a limited number of pixels over the target region. Manual ROI selection has therefore frequently been adopted in controlled thermographic studies. For example, forehead and chin ROIs were manually selected and transferred after image stabilization to analyze patient head positioning before and after non-contact tonometry [23]. Although manual or semi-automatic ROI selection is suitable for offline analysis, it is impractical for continuous autonomous monitoring because the ROI must be localized consistently without human intervention.

To overcome this limitation, several automatic facial and forehead ROI localization strategies have been investigated. Image-processing-based approaches estimate the forehead directly from thermal images using geometric cues such as head segmentation, ellipse fitting, and eye or glasses detection [8]. Landmark-based approaches instead exploit anatomical references such as eye corners, canthi, or forehead, nose, or cheek regions [24,25,26,27]. General-purpose landmark frameworks developed primarily for visible-light imagery may also provide approximate facial-region localization, although their application to low-resolution thermal imagery may be limited by the domain difference in facial appearance and available spatial detail. Multimodal approaches address this limitation by combining visible facial landmarks with thermal information, but require additional sensing and registration between modalities [15,26].

More recently, deep-learning-based approaches have been applied to thermal face detection, facial-region localization, anatomical landmark estimation, and predefined facial ROI extraction [12,28,29,30,31]. These approaches can improve automatic localization but may require face- or region-specific annotated datasets, dedicated model training, and additional computational resources. Beyond facial thermal imaging, spatial and temporal structural relationships have also been exploited to improve region segmentation under varied imaging conditions [32]. Moreover, unsupervised approaches have also been investigated to reduce dependence on explicit localization annotations. PEEKABOO [33], for example, learns context-based pixel- and shape-level representations through selective image masking without explicit bounding-box supervision. Although this demonstrates an annotation-efficient alternative for object localization, it addresses generic visual objects rather than anatomically constrained ROI localization in low-resolution thermal facial imagery.

Despite these advances, reliable automatic forehead localization remains challenging when low-resolution thermal cameras are used. Reduced facial detail can affect segmentation and landmark localization, while even small ROI displacement or boundary errors may influence the extracted temperature because relatively few pixels represent the forehead. These challenges are particularly relevant to lightweight embedded systems relying on a single low-resolution thermal camera, where methods requiring dense landmarks, multimodal registration, or dedicated forehead-specific models may be less practical. An alternative strategy is to detect a facial region that remains thermally observable under low-resolution imaging and use it as an anatomical and geometric anchor for a spatially separate ROI. The nostril region is particularly suitable for this purpose because inhaled and exhaled airflow produces localized thermal variation that facilitates detection and tracking for respiratory monitoring and respiratory rate estimation [16,17]. However, previous studies have primarily treated the nostril region as the physiological measurement site itself rather than as a geometric reference for localizing the forehead. Consequently, the use of a detected nostril bounding box to determine forehead ROI position and dimensions proportionally to the apparent nostril scale remains underexplored in low-resolution embedded thermal imaging.

To position the proposed approach within the existing literature, Table 1 summarizes representative ROI localization strategies according to their localization principle, sensing requirements, reported resolution context, anatomical or geometric basis, and embedded-deployment evidence. Motivated by this gap, the present study uses the detected nostril region as a thermally observable and anatomically related anchor for forehead ROI projection. Unlike direct forehead segmentation, dense landmark estimation, and multimodal localization, the proposed method determines the forehead ROI position and dimensions from fixed proportional geometric relationships relative to the detected nostril bounding box. This allows the ROI geometry to adapt to changes in apparent nostril scale without requiring direct forehead detection or explicit camera-distance estimation. The projected ROI is subsequently evaluated directly against manual forehead annotations before being used for forehead skin temperature estimation and real-time embedded implementation.

## 3. Method

**Figure 1:**
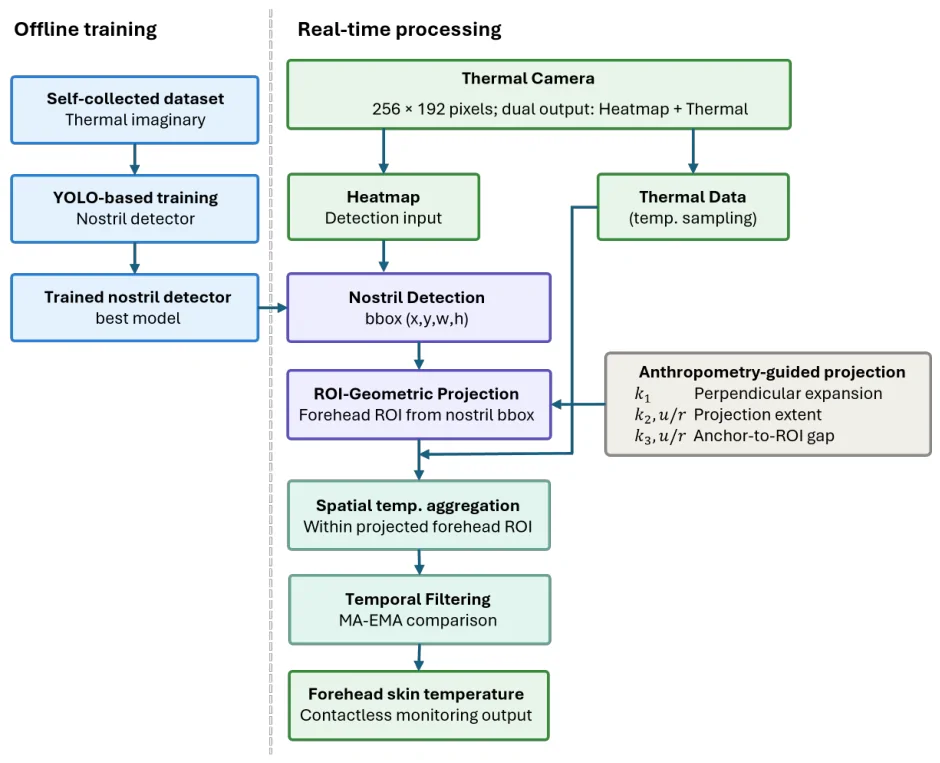
Overview of the proposed nostril-anchored forehead ROI localization and temperature-estimation framework. Nostril detection provides the geometric anchor for forehead ROI projection, followed by temperature extraction, spatial aggregation, and temporal stabilization for real-time embedded implementation.

The proposed method localizes the forehead region in low-resolution thermal images using a nostril-anchored geometric ROI projection and subsequently estimates forehead skin temperature through spatial aggregation and temporal stabilization. As illustrated in Figure 1, the framework consists of two stages: (i) offline development of a lightweight nostril detector and (ii) real-time nostril localization, geometric forehead ROI projection, and skin temperature estimation on an embedded edge device. During offline development, a YOLO-based detector is trained using a self-collected thermal dataset. During real-time operation, the trained detector localizes the nostril region, which provides the image-domain geometric anchor for the subsequent forehead ROI projection.

During real-time operation, the thermal camera acquires images at a resolution of 256×192 pixels and provides a heatmap for nostril detection together with the corresponding temperature matrix for quantitative temperature estimation. At time step *t*, the temperature data are represented as

(1) $$T^{\left(t\right)}\in R^{H\times W},$$

where *H* and *W* denote the image height and width, respectively, and $T_{i,j}^{\left(t\right)}$ represents the temperature at pixel location (i,j). The detected nostril bounding box determines the geometric reference for forehead ROI projection. Fixed orientation-dependent geometric parameters, guided by craniofacial anthropometric relationships, define the ROI dimensions and their offset relative to the nostril bounding box. Because these quantities are proportional to the apparent nostril dimensions, the projected ROI geometry adapts to changes in apparent facial scale without requiring explicit camera-distance estimation. The forehead skin temperature estimate is then obtained by spatial aggregation of the values within the projected ROI, followed by temporal stabilization to reduce frame-to-frame fluctuations. The individual processing stages are described in the following subsections.

### 3.1. Offline Training of the Nostril Detector

The proposed forehead ROI localization relies on a lightweight nostril detector trained before real-time deployment. The detected nostril region provides the image-domain geometric anchor required for subsequent forehead ROI projection. The YOLO-based nostril detector is based on the previously developed implementation reported in [34]. The principal training configuration and validation performance relevant to the present study are summarized in Table 2. The detector was trained using manually annotated thermal images containing nostril bounding boxes. Training was performed for 120 epochs using an input resolution of 640×640 pixels and a batch size of 8. Automatic data augmentation included horizontal flipping, scaling, rotation, translation, mosaic augmentation, color jitter, and blur to increase variation in nostril appearance, apparent scale, position, and image quality. The selected checkpoint was obtained at epoch 74 and achieved a validation precision of 0.978, recall of 0.994, mAP@0.5 of 0.992, and mAP@0.5:0.95 of 0.732. The selected detector remained fixed before the pilot and formal evaluations and was applied throughout all evaluations without online retraining or participant-specific adaptation.

### 3.2. Nostril Anchor Detection

**Figure 2:**
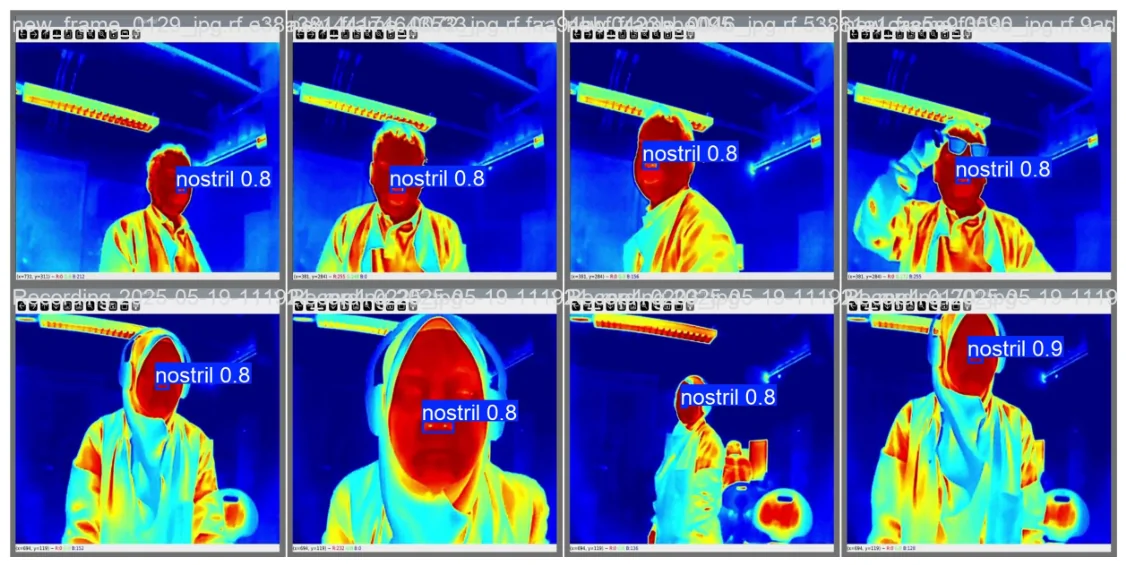
Representative nostril detection results obtained using the trained YOLO-based model under different head poses and camera-to-subject distances. The displayed thermograms are false-color visualizations provided for illustration only. Quantitative temperature estimation was performed using the corresponding temperature data rather than the displayed colors.

The detected nostril region was selected as the image-domain geometric anchor for forehead ROI projection for three reasons. First, respiratory airflow creates localized thermal contrast around the nostrils, supporting reliable detection in thermal images [16,17]. Second, the nasal and subnasal regions provide a lower-face anatomical reference with a directional relationship to the upper face supported by adult craniofacial anthropometry [18]. Third, the YOLO-based nostril detector previously developed for contactless respiratory rate monitoring using the same low-resolution thermal camera could be reused without requiring a separate forehead-specific detector [34]. The detector training dataset covered camera-to-subject distances from 0.5 m to 1.5 m. During real-time operation, the fixed detector operated without online retraining or participant-specific adaptation. Multiple nostril detections were ranked by confidence, and the highest-confidence detection was selected as the geometric anchor. Representative detection results are shown in Figure 2.

At detector-inference time step *t*, the selected nostril bounding box is represented as

(2) $$B_{n}^{\left(t\right)}=\left(x^{\left(t\right)},y^{\left(t\right)},w_{n}^{\left(t\right)},h_{n}^{\left(t\right)}\right),$$

where $\left(x^{\left(t\right)},y^{\left(t\right)}\right)$ denotes the upper-left corner of the bounding box, and $w_{n}^{\left(t\right)}$ and $h_{n}^{\left(t\right)}$ denote the width and height, respectively. The detected bounding box provides the image-domain geometric reference for subsequent forehead ROI projection. To reduce detector computation, suppress frame-to-frame bounding-box jitter, and maintain the nostril anchor during temporary missed detections, a Kalman-based ROI tracker was integrated with the YOLO detector. YOLO inference was performed every second frame. On detector-inference frames, an available highest-confidence YOLO bounding box was used as the measurement for tracker correction. On intermediate frames, the tracker predicted the nostril bounding-box position and dimensions without running YOLO. Tracker prediction was also used during temporary missed detections, provided that the target had not been declared lost. A lost-target condition reset the tracker until a new YOLO detection became available. The tracked nostril bounding box used for forehead ROI projection is represented as

(3) $${\hat{B}}_{n}^{\left(t\right)}=\left({\hat{x}}^{\left(t\right)},{\hat{y}}^{\left(t\right)},{\hat{w}}_{n}^{\left(t\right)},{\hat{h}}_{n}^{\left(t\right)}\right),$$

where the hatted variables denote tracker-estimated bounding-box coordinates and dimensions. The tracked output provides a temporally continuous geometric anchor by combining YOLO-based measurement updates with Kalman predictions, thereby reducing localization jitter and maintaining ROI continuity during intermediate frames or temporary missed detections.

### 3.3. Geometrically Anchored Forehead ROI Projection

**Figure 3:**
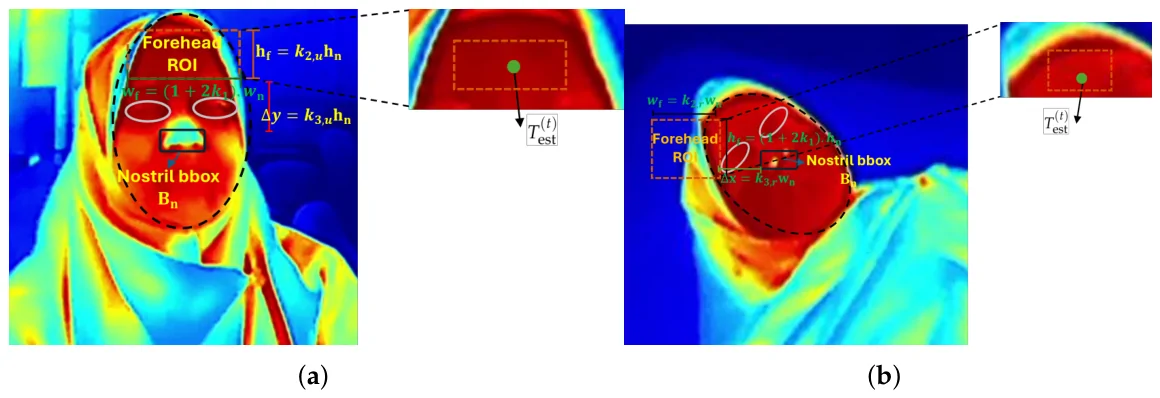
Nostril-anchored geometric forehead ROI projection under (**a**) upright and (**b**) rotated/supine configurations. In the upright configuration, the forehead ROI is projected vertically above the nostril anchor using projection extent $k_{2,u}$ and gap $k_{3,u}$. In the rotated/supine configuration, the projection axis is redirected laterally using $k_{2,r}$ and $k_{3,r}$. The coefficient $k_{1}$ controls ROI expansion perpendicular to the projection direction in both configurations.

The forehead ROI is projected from the tracked nostril bounding box, ${\hat{B}}_{n}^{\left(t\right)}$, using fixed proportional geometric parameters defined relative to the tracked nostril dimensions, as illustrate in Figure 3a for the upright configuration and Figure 3b for the rotated configuration, respectively. The ROI position, dimensions, and projection offset therefore vary with the apparent nostril scale rather than with fixed image coordinates. Two orientation-dependent configurations are defined: an upright configuration with vertical projection above the nostril region and a rotated/supine configuration with lateral projection. A common coefficient controls expansion perpendicular to the projection direction, while orientation-specific coefficients define the projection extent and anchor-to-ROI gap. Let $\left({\hat{x}}_{c}^{\left(t\right)},{\hat{y}}_{c}^{\left(t\right)}\right)$ denote the center of the tracked nostril bounding box. The coefficient $k_{1}$ controls perpendicular ROI expansion. The coefficients $k_{2,u}$ and $k_{3,u}$ define the projection extent and gap for the upright configuration, respectively, while $k_{2,r}$ and $k_{3,r}$ define the corresponding quantities for the rotated/supine configuration.

For the upright configuration, the projected forehead ROI is defined as

(4) $$\begin{array}{cc} x_{f}^{\left(t\right)}\; & ={\hat{x}}_{c}^{\left(t\right)}-\frac{1+2k_{1}}{2}{\hat{w}}_{n}^{\left(t\right)},\\ w_{f}^{\left(t\right)}\; & =\left(1+2k_{1}\right){\hat{w}}_{n}^{\left(t\right)},\\ y_{f}^{\left(t\right)}\; & ={\hat{y}}_{c}^{\left(t\right)}-\left(k_{2,u}+k_{3,u}\right){\hat{h}}_{n}^{\left(t\right)},\\ h_{f}^{\left(t\right)}\; & =k_{2,u}{\hat{h}}_{n}^{\left(t\right)}. \end{array}$$

Table 3 summarizes the geometric functions of the projection parameters. The selected parameter configuration remained fixed before the formal evaluation and was applied to all participants and experimental conditions without participant-specific geometric adjustment. Because the ROI dimensions and offsets are proportional to ${\hat{w}}_{n}^{\left(t\right)}$ and ${\hat{h}}_{n}^{\left(t\right)}$, the ROI geometry changes with apparent facial scale without requiring explicit camera-distance estimation. The upright parameters $\left(k_{1},k_{2,u},k_{3,u}\right)$ were examined during the pilot configuration stage. Projection direction is selected from the aspect ratio of the tracked nostril bounding box,

(5) $$r^{\left(t\right)}=\frac{{\hat{w}}_{n}^{\left(t\right)}}{{\hat{h}}_{n}^{\left(t\right)}}.$$

An orientation-switching threshold of $r_{\mathrm{th}}=0.9$, selected during the pilot configuration stage, determines the image-domain projection axis. A bounding box satisfying $r^{\left(t\right)}\geq r_{\mathrm{th}}$ is treated as approximately horizontal, resulting in upward forehead projection. A bounding box satisfying $r^{\left(t\right)}<r_{\mathrm{th}}$ is treated as vertically oriented, resulting in lateral projection. The aspect ratio criterion serves only as an image-domain projection heuristic and does not estimate physical yaw, pitch, or roll angles.

For the rotated/supine configuration, two candidate ROIs are generated on opposite sides of the tracked nostril center. The left-side candidate is defined as

(6) $$\begin{array}{cc} x_{f,L}^{\left(t\right)}\; & ={\hat{x}}_{c}^{\left(t\right)}-\left(k_{2,r}+k_{3,r}\right){\hat{w}}_{n}^{\left(t\right)},\\ w_{f,L}^{\left(t\right)}\; & =k_{2,r}{\hat{w}}_{n}^{\left(t\right)},\\ y_{f,L}^{\left(t\right)}\; & ={\hat{y}}_{c}^{\left(t\right)}-\frac{1+2k_{1}}{2}{\hat{h}}_{n}^{\left(t\right)},\\ h_{f,L}^{\left(t\right)}\; & =\left(1+2k_{1}\right){\hat{h}}_{n}^{\left(t\right)}. \end{array}$$

The right-side candidate is defined as

(7) $$\begin{array}{cc} x_{f,R}^{\left(t\right)}\; & ={\hat{x}}_{c}^{\left(t\right)}+k_{3,r}{\hat{w}}_{n}^{\left(t\right)},\\ w_{f,R}^{\left(t\right)}\; & =k_{2,r}{\hat{w}}_{n}^{\left(t\right)},\\ y_{f,R}^{\left(t\right)}\; & ={\hat{y}}_{c}^{\left(t\right)}-\frac{1+2k_{1}}{2}{\hat{h}}_{n}^{\left(t\right)},\\ h_{f,R}^{\left(t\right)}\; & =\left(1+2k_{1}\right){\hat{h}}_{n}^{\left(t\right)}. \end{array}$$

The rotated/supine configuration preserves proportional nostril-relative projection while redirecting the longitudinal projection axis laterally. The nostril bounding-box width defines the lateral projection dimension, whereas the bounding-box height defines perpendicular expansion. Maximum temperature within each candidate ROI is used only to resolve the unknown left–right projection direction. The candidate containing the higher maximum temperature is selected. ROI dimensions remain determined exclusively by the tracked nostril dimensions and fixed geometric parameters.

The selected ROI is clipped to the valid image boundaries such that

(8) $$0\leq x_{f}^{\left(t\right)}<x_{f}^{\left(t\right)}+w_{f}^{\left(t\right)}\leq W,\;0\leq y_{f}^{\left(t\right)}<y_{f}^{\left(t\right)}+h_{f}^{\left(t\right)}\leq H.$$

The projection design was guided by the directional relationship between the nasal/subnasal region and the upper face reported in adult craniofacial anthropometry [18]. The detected nostril bounding box, however, represents an image-domain region rather than a calibrated anatomical landmark. Accordingly, $k_{1}$, $k_{2,u}$, $k_{3,u}$, $k_{2,r}$, and $k_{3,r}$ are dimensionless geometric projection parameters rather than direct numerical conversions of anthropometric indices. Anthropometric relationships provided the geometric basis for plausible nostril-to-forehead projection, while parameter selection was performed during the pilot stage. All selected parameters remained unchanged during the formal evaluation without participant-specific adjustment.

### 3.4. Spatial Temperature Aggregation

The projected forehead ROI contains multiple temperature values whose variation may arise from sensor noise, local thermal heterogeneity, boundary contamination, and limited spatial resolution. A temperature estimate based on a single pixel can therefore be sensitive to isolated extreme values. Spatial aggregation is applied to obtain a more stable frame-level temperature estimate from the thermal data within the projected forehead ROI. Let the set of temperature values within the projected forehead ROI at time *t* be

(9) $$\Omega^{\left(t\right)}=\left\{T_{i,j}^{\left(t\right)}|\left(i,j\right)\in F^{\left(t\right)}\right\},$$

where $F^{\left(t\right)}$ denotes the set of pixel coordinates belonging to the projected forehead ROI. The frame-level forehead temperature estimate is represented as

(10) $$T_{\mathrm{est}}^{\left(t\right)}=A\;\left(\Omega^{\left(t\right)}\right),$$

where A(·) denotes the spatial aggregation operator.

**Figure 4:**
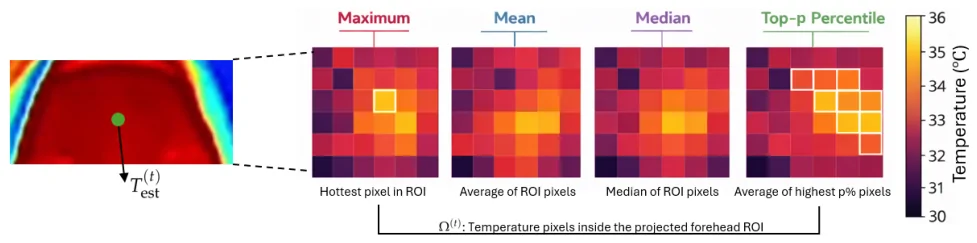
Spatial aggregation strategies evaluated within the projected forehead ROI. Maximum aggregation selects the highest temperature value, mean and median aggregation summarize the complete ROI temperature distribution, and $P_{95}$ aggregation selects the 95th percentile of the ROI temperature distribution. The white frames indicate the pixels selected within the Top-*P* percentile for temperature estimation.

Four candidate spatial aggregation operators were compared during the pilot configuration stage: maximum, mean, median, and 95th-percentile ($P_{95}$) aggregation. Selection was based on downstream temperature-estimation performance relative to the practical forehead temperature comparator. Figure 4 illustrates the four candidate aggregation strategies.

The maximum aggregation strategy is defined as

(11) $$T_{\max}^{\left(t\right)}=\max\;\left(\Omega^{\left(t\right)}\right),$$

where the maximum operator preserves the highest ROI temperature but remains sensitive to isolated hot pixels, sensor noise, and other extreme values.

The mean aggregation strategy is defined as

(12) $$T_{\mathrm{mean}}^{\left(t\right)}=\frac{1}{|\Omega^{\left(t\right)}|}\sum_{T_{i,j}^{\left(t\right)}\in \Omega^{\left(t\right)}}T_{i,j}^{\left(t\right)},$$

where all temperature values within the projected ROI contribute equally to the frame-level estimate. Cooler values near the ROI boundaries can therefore influence the resulting temperature.

The median aggregation strategy is defined as

(13) $$T_{\mathrm{median}}^{\left(t\right)}=\mathrm{median}\;\left(\Omega^{\left(t\right)}\right),$$

providing greater robustness to isolated extreme values while representing the central tendency of the ROI temperature distribution.

The percentile-based aggregation operator is generally defined as

(14) $$T_{P_{p}}^{\left(t\right)}={\mathrm{Percentile}}_{p}\;\left(\Omega^{\left(t\right)}\right),$$

where p∈[0,100] denotes the percentile level. For the pilot comparison, the percentile-based candidate was configured at p=95, resulting in

(15) $$T_{P_{95}}^{\left(t\right)}={\mathrm{Percentile}}_{95}\;\left(\Omega^{\left(t\right)}\right).$$

The $P_{95}$ operator emphasizes the warmer portion of the ROI while reducing dependence on a single hottest pixel compared with maximum aggregation. Reduced contribution from cooler ROI values can also limit the influence of boundary contamination compared with mean or median aggregation. However, $P_{95}$ aggregation does not explicitly segment or exclude hair, eyebrows, facial edges, or background pixels and therefore does not guarantee exclusive sampling of anatomically defined forehead skin. Performance comparison among the four candidate operators was conducted during the pilot configuration stage using temperature-estimation error as the selection criterion. The best-performing aggregation configuration was subsequently fixed before the formal evaluation.

### 3.5. Temporal Stabilization

The frame-level forehead temperature estimate, $T_{\mathrm{est}}^{\left(t\right)}$, may exhibit short-term fluctuations caused by sensor noise, minor head motion, and pixel-level variability. Two causal temporal filters were evaluated during the pilot configuration stage: moving average (MA) and exponential moving average (EMA). Filter performance was compared using variance reduction to quantify suppression of temporal fluctuations and mean sample age to characterize the temporal weighting of recent and older observations. Candidate MA window sizes of M=5, 10, and 15 and EMA coefficients of α=0.3, 0.2, and 0.1 were evaluated. The best-performing filter configuration was selected during the pilot stage and subsequently fixed before the formal evaluation.

The MA filter computes the average of the most recent *M* observations,

(16) $${\tilde{T}}_{\mathrm{MA}}^{\left(t\right)}=\frac{1}{M}\sum_{m=0}^{M-1}T_{\mathrm{est}}^{\left(t-m\right)},$$

where *M* denotes the smoothing window size. Increasing *M* increases the contribution of older observations and generally provides stronger suppression of short-term fluctuations at the cost of lower temporal responsiveness.

The EMA filter recursively updates the temperature estimate according to

(17) $${\tilde{T}}_{\mathrm{EMA}}^{\left(t\right)}=\alpha T_{\mathrm{est}}^{\left(t\right)}+\left(1-\alpha \right){\tilde{T}}_{\mathrm{EMA}}^{\left(t-1\right)},$$

where α∈(0,1) denotes the smoothing coefficient. A larger α assigns greater weight to the current observation and provides higher temporal responsiveness, whereas a smaller α assigns greater weight to previous observations and produces stronger smoothing. The recursive formulation requires only the current frame-level estimate and the previous smoothed estimate, reducing memory and computational requirements for real-time embedded implementation.

Variance reduction (VR) quantifies suppression of temporal fluctuations and is calculated as

(18) $$\mathrm{VR}\;\left(\%\right)=\left(1-\frac{\sigma_{\mathrm{smooth}}^{2}}{\sigma_{\mathrm{raw}}^{2}}\right)\times 100,$$

where $\sigma_{\mathrm{raw}}^{2}$ and $\sigma_{\mathrm{smooth}}^{2}$ denote the temporal variances before and after smoothing, respectively.

Temporal weighting was additionally characterized by the mean sample age, defined as the center of mass of the corresponding causal weighting kernel. For the MA filter, the mean sample age in frames is

(19) $$A_{\mathrm{MA}}=\frac{M-1}{2}.$$

For the EMA filter, the mean sample age is

(20) $$A_{\mathrm{EMA}}=\frac{1-\alpha }{\alpha }.$$

The mean sample age represents the average age of observations contributing to the filtered estimate according to the corresponding weighting kernel. The metric provides a descriptive measure of temporal weighting and does not represent the actual signal-dependent temporal lag of either filter. A larger VR indicates stronger suppression of frame-to-frame fluctuations, whereas a larger mean sample age indicates greater weighting of older observations and lower temporal responsiveness.

## 4. Experimental Evaluations and Results

### 4.1. Experimental Setup and Evaluation Protocol

The evaluation was designed as a controlled module-level feasibility study addressing three aspects of the proposed framework: (i) direct spatial localization of the projected forehead ROI, (ii) application-level forehead skin temperature estimation, and (iii) real-time embedded implementation under the evaluated monitoring conditions. A TOPDON TC001 low-resolution thermal camera was used to acquire thermal data. The principal camera specifications are summarized in Table 4. The camera was operated using the factory calibration without additional blackbody-based or traceable calibration. The camera provided a heatmap and corresponding thermal data. The heatmap was used for nostril detection and visualization, whereas the thermal data was used for forehead temperature extraction and spatial aggregation. Temperature calculations were therefore based on the thermal data rather than the displayed heatmap colors.

A commercially available medical non-contact infrared forehead thermometer (SPIT003, Guangdong Shipai Technology Co., Ltd., Foshan, China; device-labelled measurement range: 32.0–42.9 °C; measurement distance: 3–5cm; measurement time: approximately 1 s) was used as a practical forehead temperature comparator. Selection of the comparator was based on the common forehead measurement target, rapid non-contact operation, and practical use across the evaluated postures and experimental conditions. The handheld thermometer and thermal-camera method sampled approximately the same central forehead region but used different sensing principles and spatial sampling areas. The handheld thermometer was therefore not treated as a gold-standard or traceably calibrated reference. The temperature-related results consequently represent agreement with the practical comparator under the evaluated conditions rather than absolute clinical or metrological accuracy. The common experimental conditions, participant preparation, measurement sequence, and trial-processing procedure are summarized in Table 5.

The evaluation comprised separate pilot and formal stages. The pilot stage included two healthy volunteers excluded from the formal participant cohort, and supported selection of the geometric projection configuration, spatial aggregation operator, and temporal stabilization configuration. The selected processing configuration was fixed before formal evaluation, without participant- or condition-specific parameter adjustment. The formal stage included ten independent participants. Direct spatial localization was assessed using five temporally separated annotated frames for each participant at camera-to-subject distances of 0.5, 1.0, and 1.5 m, resulting in 150 annotated frames. The formal spatial annotations were used only for evaluation and were not used for geometric parameter adjustment. Application-level temperature evaluation comprised nine acquisition blocks per participant: two baseline-seated blocks, three distance blocks, two head pose blocks, and two supine blocks, resulting in 90 paired trial-level temperature measurements. For each acquisition block, the trial-level thermal estimate was calculated from the mean of the temporally stabilized samples following identification of the steady-state segment. The same 90 paired trial-level measurements were used for calculation of MAE, RMSE, bias, and Bland–Altman agreement. Sequential acquisition by the handheld comparator and thermal camera, together with differences in spatial sampling, limited interpretation to practical measurement agreement following the fixed offset adjustment under the evaluated indoor conditions. Measurement uncertainty from both devices contributes to the observed differences.

Bias was defined as the mean signed difference between the trial-level thermal estimate and the corresponding handheld comparator measurement:

(21) $$\mathrm{Bias}=\frac{1}{N}\sum_{i=1}^{N}\left({\bar{T}}_{\mathrm{est},i}-T_{\mathrm{cmp},i}\right),$$

where *N* denotes the number of paired trial-level measurements, ${\bar{T}}_{\mathrm{est},i}$ denotes the mean steady-state thermal estimate for trial *i*, and $T_{\mathrm{cmp},i}$ denotes the corresponding handheld thermometer measurement. Negative bias indicates underestimation relative to the comparator, whereas positive bias indicates overestimation. Unless otherwise stated, all temperature-related error metrics are reported in °C.

### 4.2. Method Selection and Processing Configuration

A pilot study was conducted before the formal evaluation to select three processing components: the forehead ROI method, spatial temperature aggregation operator, and temporal stabilization filter. Two healthy volunteers participated in the pilot stage and were excluded from the formal participant cohort. Selection of the complete processing configuration was completed before the formal evaluation, without participant- or condition-specific tuning. The proposed nostril-anchored projection was compared with a fixed image-frame crop and a MediaPipe Pose-based landmark method [35]. MediaPipe Pose was included as a lightweight cross-domain landmark baseline to assess forehead ROI estimation without thermal-domain-specific training. Implementation details for the three candidate methods are summarized in Table 6.

**Figure 5:**
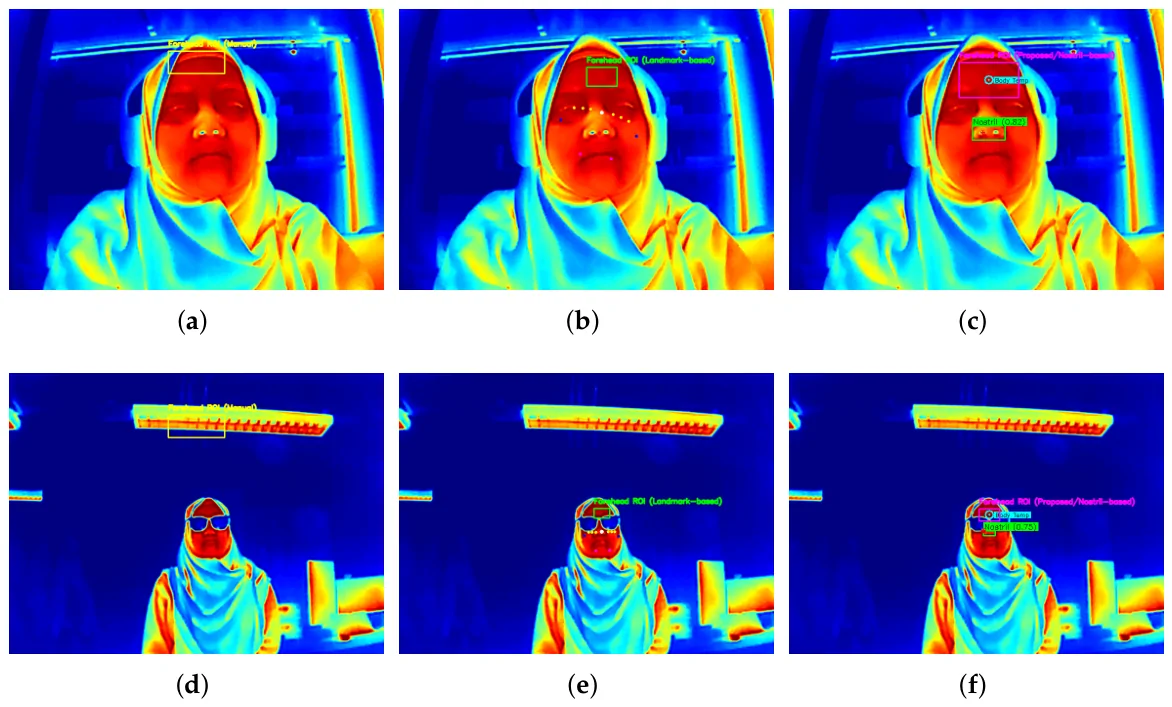
Representative comparison of (**a**,**d**) the fixed image-frame crop, (**b**,**e**) the MediaPipe Pose-based ROI, and (**c**,**f**) the proposed nostril-anchored projection. The upper row shows a closer frontal condition, whereas the lower row shows an additional qualitative stress condition at approximately 2 m with the participant wearing glasses. The additional stress condition was included for illustration and was not part of the formal distance protocol. The gray box indicates the fixed image-frame ROI, the green box indicates the MediaPipe Pose-based ROI, and, for the proposed method, the green box indicates the detected nostril ROI while the magenta box indicates the projected forehead ROI. The displayed heatmaps are provided for visualization; all quantitative temperature calculations used the corresponding temperature data.

Figure 5 presents representative qualitative examples of the three candidate ROI methods. The examples were obtained from one representative pilot participant. Under the closer frontal condition, all three methods produced an approximate forehead region. Under the additional stress condition, the fixed crop could not account for changes in facial position and apparent scale, while the cross-domain landmark method showed reduced localization consistency under lower facial detail and partial occlusion. The nostril-anchored method adjusted ROI position and dimensions according to the detected nostril region.

**Figure 6:**
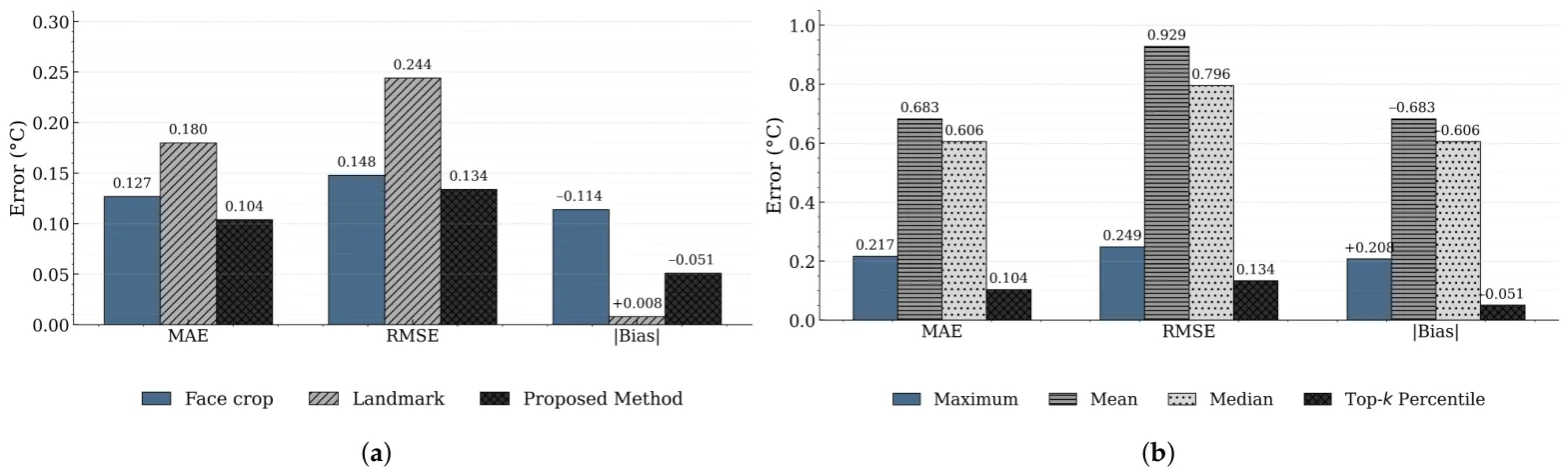
Pilot comparison of (**a**) candidate ROI methods and (**b**) spatial temperature aggregation operators using MAE, RMSE, and |Bias|. Bias bar heights represent absolute magnitudes, whereas the annotations report the corresponding signed values.

ROI method selection during the pilot stage was based on downstream temperature-estimation error relative to the practical forehead temperature comparator. As shown in Figure 6a, the proposed ROI method produced an MAE of 0.104 °C, RMSE of 0.134 °C, and bias of −0.051 °C. The fixed crop produced an MAE of 0.127 °C, RMSE of 0.148 °C, and bias of −0.114 °C. The MediaPipe Pose-based method produced an MAE of 0.180 °C, RMSE of 0.244 °C, and bias of +0.008 °C. The near-zero signed bias for the landmark method occurred alongside a larger MAE and RMSE, indicating cancellation between positive and negative errors. The pilot temperature-error comparison supported the selection of the nostril-anchored ROI for subsequent processing. The pilot comparison was not treated as direct evidence of spatial localization accuracy. Direct annotation-based forehead ROI localization was evaluated separately using spatial localization metrics in the subsequent evaluation. Following selection of the nostril-anchored ROI, four spatial aggregation operators were compared within the projected forehead region: maximum, mean, median, and $P_{95}$. Figure 6b shows the corresponding temperature-estimation errors. The maximum operator showed greater sensitivity to isolated hot pixels, whereas mean and median aggregation incorporated the complete ROI temperature distribution and therefore received greater contribution from cooler ROI values.

The $P_{95}$ operator produced the lowest pilot error, with an MAE of 0.104 °C, RMSE of 0.134 °C, and bias of −0.051 °C. Consequently, $P_{95}$ was selected for the subsequent processing configuration. The selected operator emphasizes the warmer portion of the ROI while reducing dependence on a single hottest pixel. The $P_{95}$ operation represents an engineering approach for reducing sensitivity to ROI-boundary and pixel-level variability rather than a physiological model of forehead temperature distribution.

The final pilot comparison evaluated temporal stabilization. Candidate MA and EMA configurations were compared using variance reduction and mean sample age, as summarized in Table 7. Increasing smoothing strength produced greater variance reduction together with a larger mean sample age. EMA with α=0.3 was selected because the configuration provided greater variance reduction than MA with M=5 while retaining a comparably low mean sample age. The recursive EMA formulation also required only the current frame-level estimate and the previous smoothed estimate. The final processing configuration comprised the proposed nostril-anchored ROI projection, $P_{95}$ spatial aggregation, and EMA temporal stabilization with α=0.3. The selected configuration remained fixed before the formal evaluation and was applied to all formal participants and experimental conditions without further adjustment. The pilot results therefore serve as configuration-selection evidence rather than overall performance metrics for the complete system.

#### 4.2.1. Exploratory Sensitivity Analysis of the Geometric Projection Coefficients

**Figure 7:**
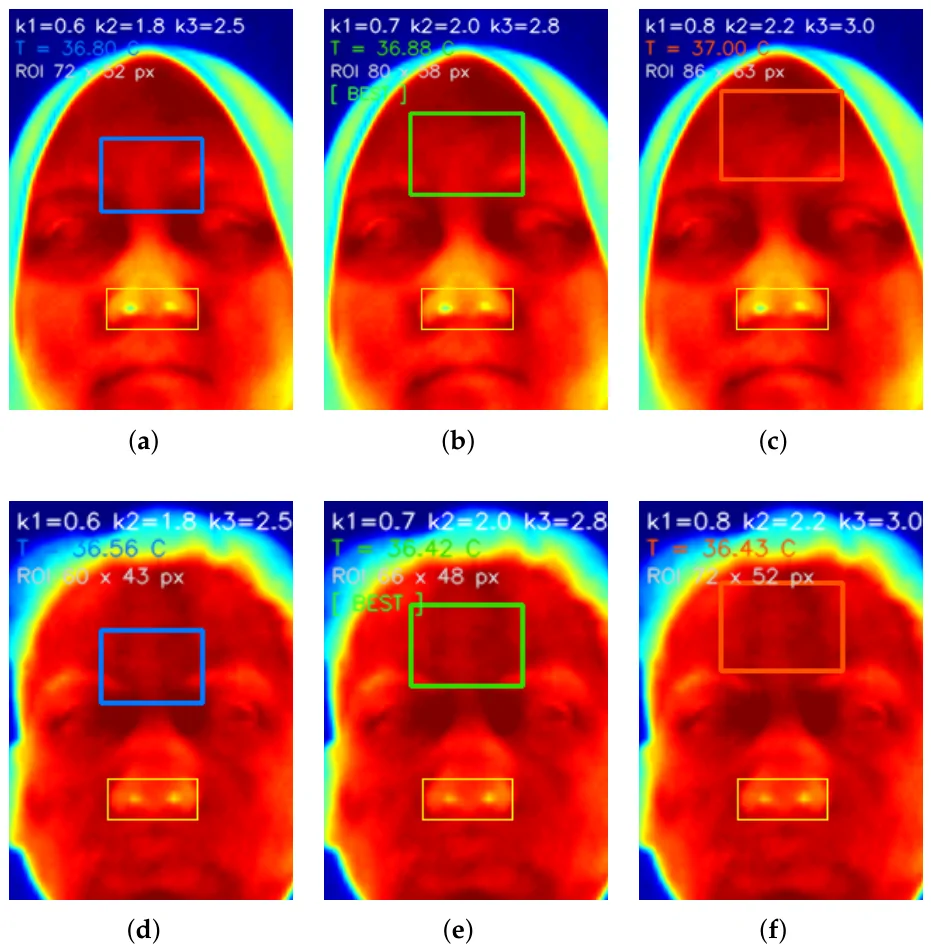
Exploratory local sensitivity analysis of the upright projection configuration using baseline recordings from two pilot participants, (**a**–**c**) correspond to Participant 1, whereas (**d**–**f**) correspond to Participant 2. For both participants, (**a**,**d**), (**b**,**e**), and (**c**,**f**) show the upright parameter configurations $\left(k_{1},k_{2,u},k_{3,u}\right)=\left(0.6,1.8,2.5\right)$, (0.7,2.0,2.8), and (0.8,2.2,3.0), respectively. Increasing the geometric coefficients increased the projected ROI dimensions while maintaining nostril-relative geometric placement. All remaining processing stages were unchanged.

An exploratory local sensitivity analysis was conducted for the upright projection configuration to examine changes in forehead ROI geometry and temperature-estimation performance under moderate variation in the geometric coefficients. Baseline recordings from both independent pilot participants were used to minimize variation associated with camera distance, head orientation, and posture. Three upright parameter configurations, $\left(k_{1},k_{2,u},k_{3,u}\right)$, were evaluated. Nostril detection, $P_{95}$ spatial aggregation, and EMA temporal stabilization with α=0.3 remained unchanged throughout the analysis. Figure 7 illustrates the resulting ROI geometry for the three evaluated configurations. As shown in Figure 7 and quantified in Table 8, increasing the geometric coefficients produced larger projected ROI dimensions, while temperature-estimation errors remained within a relatively narrow range. MAE ranged from 0.726 to 0.751 °C, and RMSE ranged from 0.817 to 0.848 °C. The nominal upright configuration, $\left(k_{1},k_{2,u},k_{3,u}\right)=\left(0.7,2.0,2.8\right)$, represents the intermediate geometric configuration among the evaluated perturbations rather than an error-minimizing optimum. The observed error range supports local robustness to the evaluated coefficient perturbations but does not establish global parameter optimality.

#### 4.2.2. Direct Spatial Evaluation of Forehead ROI Localization

A direct spatial evaluation was performed after completion of the processing configuration selection. The pilot spatial evaluation used the two independent pilot participants at camera-to-subject distances of 0.5, 1.0, and 1.5 m. Twenty temporally separated frames were annotated for each participant at each distance, resulting in 120 annotated frames (2 participants × 3 distances × 20 frames). Frame selection was performed after the initial warm-up period with temporal separation to reduce redundancy between consecutive images. The rectangular manual annotation represented visible central forehead skin above the eyebrows and below the hairline, excluding eyebrows, eyes, hair, and background. Manual annotations were created after processing-configuration selection and were not used for geometric parameter adjustment. Three ROI localization methods were evaluated using the same annotated frames: the proposed nostril-anchored projection with $\left(k_{1},k_{2,u},k_{3,u}\right)=\left(0.7,2.0,2.8\right)$, the fixed image-frame crop, and the MediaPipe Pose-based cross-domain landmark baseline. Let $F_{m}^{\left(t\right)}$ denote the ROI generated by method *m* at frame *t*, and let $G^{\left(t\right)}$ denote the corresponding manually annotated forehead region.

Spatial overlap was evaluated using IoU,

(22) $${\mathrm{IoU}}_{m}^{\left(t\right)}=\frac{\left|F_{m}^{\left(t\right)}\cap G^{\left(t\right)}\right|}{\left|F_{m}^{\left(t\right)}\cup G^{\left(t\right)}\right|},$$

where a higher value indicates greater overlap between the generated ROI and the manual annotation. The FIR was defined as

(23) $${\mathrm{FIR}}_{m}^{\left(t\right)}=\frac{\left|F_{m}^{\left(t\right)}\cap G^{\left(t\right)}\right|}{\left|F_{m}^{\left(t\right)}\right|},$$

where a higher FIR indicates that a larger proportion of the generated ROI lies within the manually annotated forehead region. The NCE was calculated as

(24) $${\mathrm{NCE}}_{m}^{\left(t\right)}=\frac{{∥c_{F_{m}}^{\left(t\right)}-c_{G}^{\left(t\right)}∥}_{2}}{\sqrt{{\left(w_{G}^{\left(t\right)}\right)}^{2}+{\left(h_{G}^{\left(t\right)}\right)}^{2}}},$$

where $c_{F_{m}}^{\left(t\right)}$ and $c_{G}^{\left(t\right)}$ denote the centers of the generated and manually annotated regions, respectively, and $w_{G}^{\left(t\right)}$ and $h_{G}^{\left(t\right)}$ denote the dimensions of the manual annotation. A lower NCE indicates closer alignment between the two region centers.

**Figure 8:**
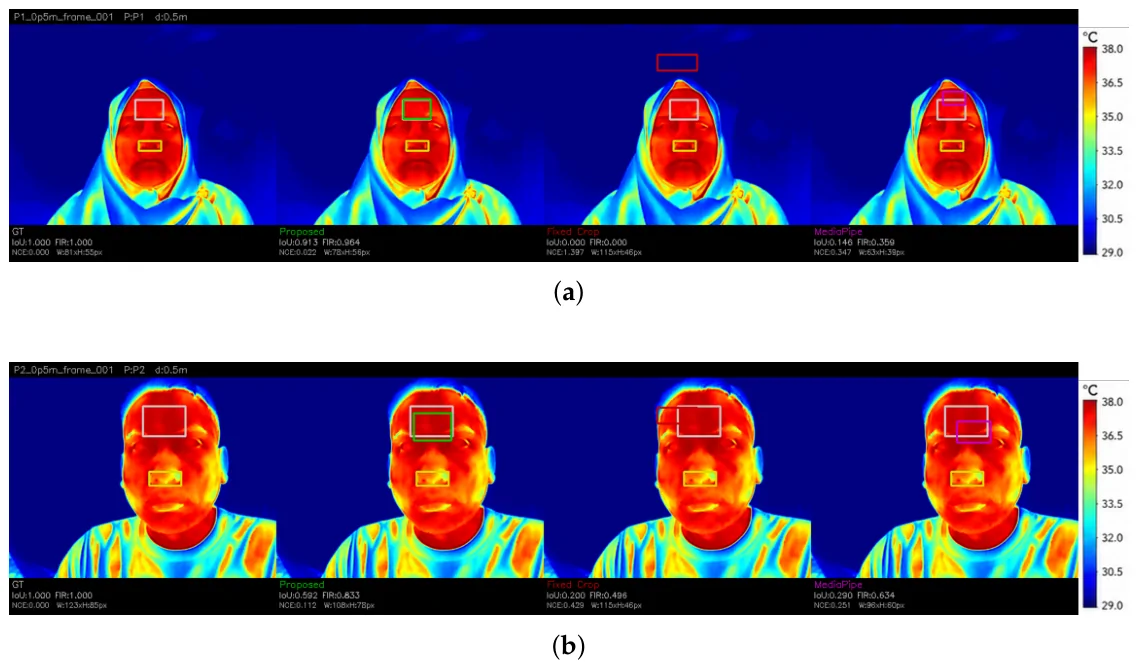
Representative direct spatial comparisons for (**a**) Participant 1 and (**b**) Participant 2 at a camera-to-subject distance of 0.5 m. Each panel shows, from left to right, the manually annotated forehead region, proposed nostril-anchored projection, fixed image-frame crop, and MediaPipe Pose-based ROI. The white box indicates the manually annotated reference forehead ROI, the green box indicates the proposed projected forehead ROI, the dark red box indicates the fixed image-frame ROI, and the magenta box indicates the MediaPipe Pose-based ROI. The yellow box indicates the detected nostril region used as the image-domain geometric anchor. The corresponding IoU, FIR, and NCE values are displayed for each localization method. The yellow box indicates the detected nostril region used as the image-domain geometric anchor. The displayed heatmaps are provided only for visualization; all spatial metrics were calculated from the corresponding ROI coordinates.

For descriptive comparison, a frame was additionally classified as a spatial failure when the IoU was below 0.30. The threshold was used only to summarize substantial localization mismatch and was not used for parameter selection or tuning. Pilot IoU, FIR, and NCE values are reported as mean ± standard deviation across the annotated frames. Representative spatial localization examples are presented in Figure 8, while the overall quantitative comparison across all 120 annotated pilot frames is summarized in Table 9.

The proposed projection produced the highest mean IoU (0.603±0.150) and FIR (0.704±0.161) and the lowest mean NCE (0.146±0.138) among the three evaluated methods. Only one of the 120 proposed-method frames met the descriptive spatial-failure criterion, corresponding to a failure rate of 0.8%. The fixed image-frame crop showed substantially lower spatial correspondence, with a 100% failure rate, consistent with the absence of adaptation to facial position and apparent scale. The MediaPipe Pose-based baseline achieved intermediate spatial performance but showed greater localization variability under the low-resolution thermal-domain input. The MediaPipe result represents the performance of a cross-domain landmark baseline rather than a thermal-specific localization method.

Table 10 summarizes the distance-dependent spatial performance of the proposed method during the pilot evaluation. The highest spatial correspondence occurred at 0.5 m, with an IoU of 0.73±0.10, FIR of 0.84±0.08, and NCE of 0.08±0.04. Lower spatial correspondence was observed at 1.0 and 1.5 m. Nostril detection was obtained in all evaluated frames, while mean nostril IoU decreased from 0.80 at 0.5 m to 0.75 at 1.5 m. The distance-dependent results therefore indicate stronger spatial localization at the closest evaluated distance and reduced performance at the two farther distances. To assess generalization beyond the pilot participants, an additional annotation-based spatial validation was performed using the distance-condition recordings from all ten formal participants. Five temporally separated frames were selected for each participant at each camera-to-subject distance of 0.5, 1.0, and 1.5 m, resulting in 150 manually annotated frames (10 participants × 3 distances × 5 frames). The same manual forehead definition, IoU, FIR, NCE, and descriptive spatial-failure criterion used in the pilot evaluation were retained. Geometric projection parameters remained fixed at the configuration selected before the formal evaluation, and the formal-cohort annotations were not used for parameter adjustment or participant-specific tuning. The formal-cohort analysis evaluated only the proposed nostril-anchored projection because the purpose was assessment of generalization of the selected method beyond the pilot participants. Comparative evaluation with the fixed crop and MediaPipe Pose-based baseline remained confined to the independent pilot stage.

Table 11 summarizes direct spatial localization performance of the proposed method across the ten-participant formal cohort. The overall participant-level mean IoU was 0.714, FIR was 0.870, and NCE was 0.091. Only one of the 150 individual frames met the descriptive spatial-failure criterion, corresponding to a failure rate of 0.7%. The strongest spatial localization was observed at 0.5 m. Mean IoU was lower but identical at 1.0 and 1.5 m, whereas FIR and NCE did not follow a strictly monotonic distance-dependent pattern. The 1.0 m condition showed the largest NCE variability and contained the single spatial failure. Participant-level overall mean IoU ranged from 0.641 to 0.772 across the ten formal participants, FIR ranged from 0.794 to 0.951, and NCE ranged from 0.066 to 0.159. The formal-cohort results demonstrate that spatial localization performance extended beyond the two pilot participants under the three evaluated frontal distance conditions. Direct annotation-based validation under quantitatively defined head orientations and supine posture was not included in the formal spatial evaluation.

### 4.3. Application-Level Temperature Estimation Performance

**Figure 9:**
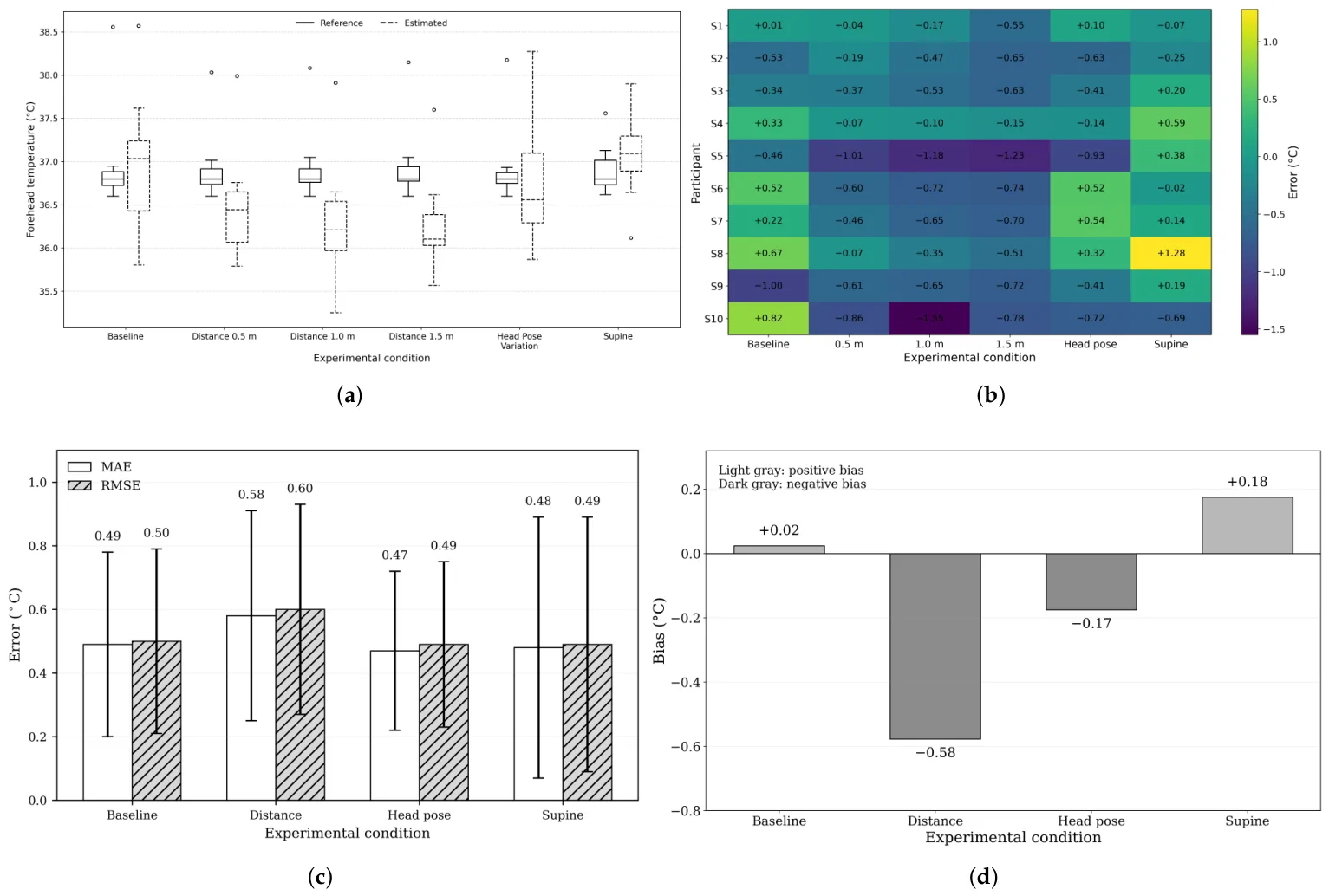
Application-level forehead temperature-estimation performance across ten participants and the evaluated experimental conditions: (**a**) distributions of the medical infrared forehead thermometer measurements and estimated forehead temperatures, (**b**) signed estimation error map calculated as ${\bar{T}}_{\mathrm{est},i}-T_{\mathrm{cmp},i}$, (**c**) condition-level MAE and RMSE with standard deviations across participants, and (**d**) condition-level mean bias. Positive values indicate overestimation, while negative values indicate underestimation relative to the forehead temperature comparator.

Using the selected processing configuration, forehead temperature estimation was evaluated across all ten formal participants and experimental conditions. The evaluation comprised 90 paired trial-level measurements obtained using the fixed nostril-anchored ROI projection, $P_{95}$ spatial aggregation, and EMA temporal stabilization with α=0.3. Across all measurements, the proposed method achieved an overall MAE of 0.514 °C, RMSE of 0.614 °C, and mean signed difference of −0.187 °C. The preceding direct spatial evaluation quantified correspondence between the projected ROI and manually annotated forehead regions. The present analysis instead evaluates downstream forehead temperature estimates obtained from the selected ROIs. The reported errors therefore quantify application-level agreement with the medical non-contact infrared forehead thermometer rather than direct ROI localization accuracy. Figure 9 summarizes the participant- and condition-level temperature-estimation results. Figure 9a compares the distributions of the handheld thermometer measurements and thermal-camera estimates. Figure 9b shows the signed errors, calculated as the thermal estimate minus the handheld thermometer measurement. Figure 9c,d summarize the corresponding condition-level MAE, RMSE, and mean bias.

The detailed participant-level results are reported in Table 12. All participants showed negative bias under each of the three distance conditions. For most participants, the magnitude of underestimation increased as the camera-to-subject distance increased, indicating a systematic distance-related effect rather than randomly directed errors. The largest absolute difference was observed for S10 at 1.5 m, where the estimated temperature was 1.65 °C lower than the handheld thermometer measurement. In contrast, the baseline, head pose, and supine conditions showed both positive and negative errors, indicating greater inter-participant variability in error direction.

The condition-level results averaged across participants are summarized in Table 13. The mean MAE ranged from 0.47 to 0.58 °C. The head pose condition produced the lowest mean MAE (0.47 °C), followed by the supine (0.48 °C) and baseline (0.49 °C) conditions. The combined distance condition produced the highest mean MAE (0.58 °C), highest mean RMSE (0.60 °C), and strongest negative bias (−0.58 °C). The baseline condition showed the mean bias closest to zero (+0.02 °C). The temperature-estimation pipeline remained operational under all evaluated conditions. Nevertheless, the consistently negative errors observed in the distance trials indicated that increasing camera-to-subject distance remained an important source of application-level measurement variation. This pattern is consistent with the direct spatial evaluation, where localization performance was strongest at 0.5 m and lower at the two farther distances. Formal-cohort spatial validation across all ten participants further showed that the distance-related spatial behavior was not strictly monotonic across all localization metrics. Accordingly, geometric adaptation to apparent nostril scale should not be interpreted as distance-invariant localization or temperature-estimation performance.

**Figure 10:**
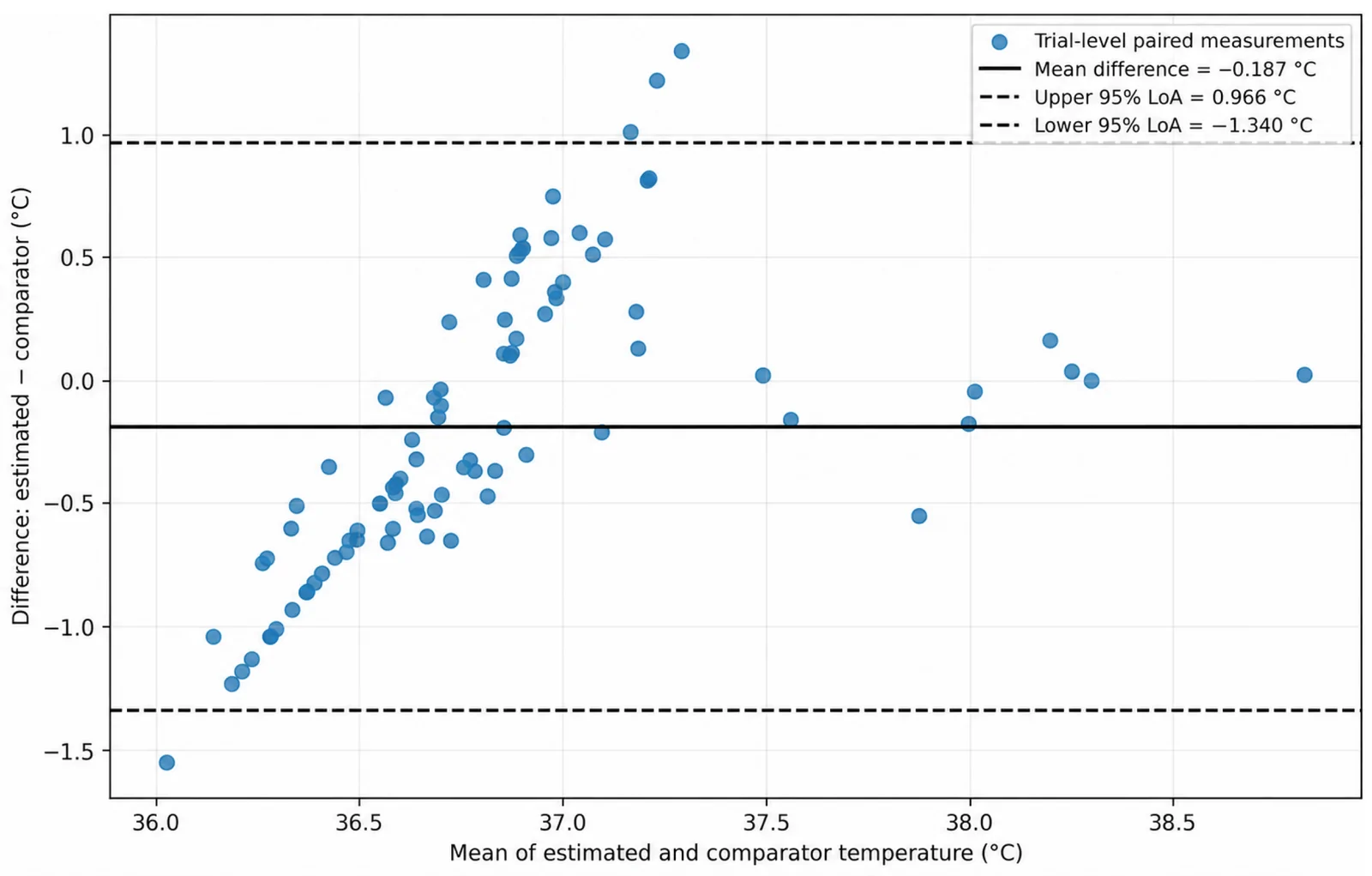
Bland–Altman analysis of the agreement between the proposed forehead skin temperature estimates and the medical non-contact infrared forehead thermometer measurements. The solid line indicates the mean difference, and the dashed lines indicate the 95% limits of agreement, calculated as the mean difference ±1.96 SD. The mean difference was −0.187 °C, with limits of agreement ranging from −1.340 °C to 0.966 °C.

Figure 10 presents the Bland–Altman analysis using the same 90 paired trial-level measurements included in the overall error analysis. The mean difference was −0.187 °C, indicating a small overall tendency for the proposed thermal method to underestimate the handheld thermometer measurement. The 95% limits of agreement ranged from −1.340 °C to 0.966 °C, showing that the magnitude and direction of individual trial differences remained variable despite the relatively small overall mean bias. These results characterize agreement between the two forehead temperature measurement approaches under the evaluated controlled indoor conditions. The results do not represent validation of absolute clinical or core-body temperature accuracy because the handheld thermometer served as a practical comparator rather than a traceably calibrated metrological or clinical reference.

### 4.4. Real-Time Embedded Performance

**Figure 11:**
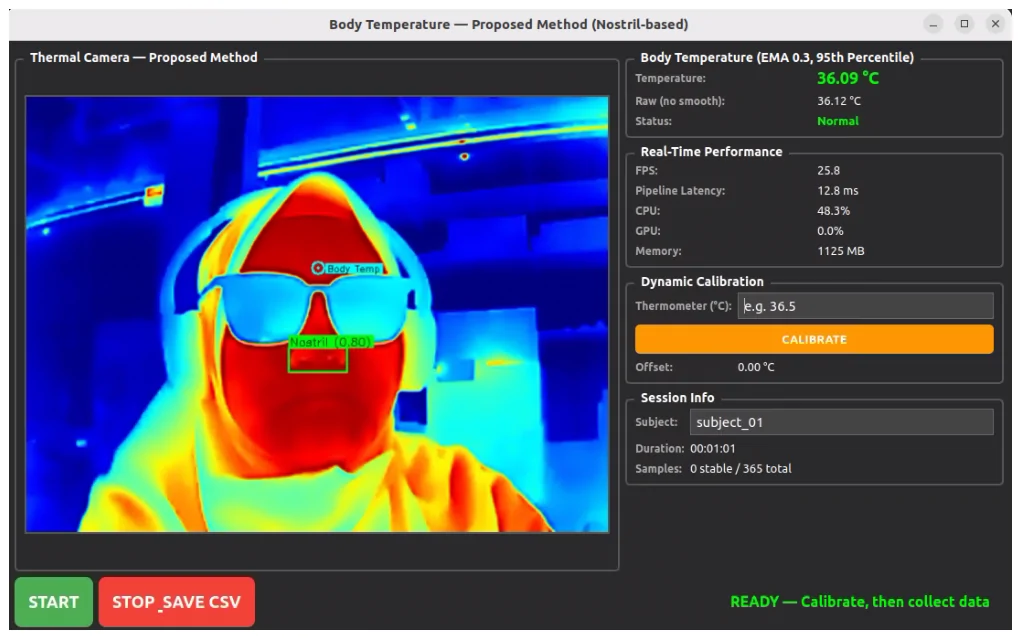
Real-time graphical user interface of the proposed forehead temperature-estimation system, displaying the live heatmap, nostril detection, projected forehead ROI, temperature estimates, and system performance indicators. The displayed heatmap is used for visualization and nostril detection, whereas quantitative temperature estimation uses the corresponding temperature data.

The proposed method was deployed and evaluated on an Jetson Orin Nano Developer Kit (NVIDIA Corporation, Santa Clara, CA, USA; 6-core ARM Cortex-A78AE CPU, 8 GB LPDDR5 RAM) running Ubuntu 20.04 LTS. to assess suitability for real-time embedded implementation. Figure 11 shows the graphical user interface integrating the live heatmap, nostril detection, projected forehead ROI, frame-level and EMA-smoothed temperature estimates, session information, and runtime indicators. Online execution of the complete processing pipeline demonstrates real-time embedded operation rather than offline post-processing.

Runtime performance was evaluated after the initial warm-up period during stable operation. The results are summarized in Table 14. A total of 4788 frames were processed over 198.5 s, producing an average throughput of 24.12±1.43 FPS, close to the nominal 25 Hz acquisition rate of the thermal camera. The mean, median, and 95th-percentile system latencies were 48.07, 60.53, and 95.98 ms, respectively. The sustained throughput reflects online pipelined processing and is therefore distinct from the latency associated with an individual processing cycle. Average CPU and GPU utilization were 36.85% and 22.46%, respectively, with transient GPU utilization reaching 99.50%. Memory consumption was approximately 1.10 GB. Runtime profiling showed an average geometric forehead ROI projection time of 3.79 ms per frame, corresponding to approximately 7.9% of the measured mean system latency. The limited computational contribution of the geometric projection supports real-time embedded operation on the NVIDIA Jetson Orin Nano.

## 5. Discussion

The results support the feasibility of nostril-anchored geometric projection for forehead ROI localization and subsequent forehead skin temperature estimation using a single low-resolution thermal camera. The evaluation separately examined spatial localization, downstream temperature estimation, and real-time embedded implementation, allowing localization performance to be distinguished from application-level temperature agreement. During the pilot evaluation, the proposed nostril-anchored projection showed higher spatial correspondence with manually annotated forehead regions than the fixed image-frame crop and MediaPipe Pose-based cross-domain landmark baseline. The pilot comparison involved 120 manually annotated frames from two independent participants and supported selection of the nostril-anchored ROI for subsequent processing. Formal-cohort spatial validation extended the analysis to all ten participants used in the application-level evaluation. Across the ten participant-level means derived from 150 balanced annotated frames, the proposed method achieved an overall IoU of 0.714, FIR of 0.870, and NCE of 0.091. Only one of the 150 individual frames met the descriptive spatial-failure criterion, corresponding to a failure rate of 0.7%. The formal-cohort results therefore reduce the uncertainty associated with extrapolating localization performance from the two pilot participants to the complete formal cohort under the evaluated frontal distance conditions.

The geometric formulation determines ROI position and dimensions proportionally from the tracked nostril bounding box, allowing ROI geometry to respond to changes in apparent facial scale without explicit camera-distance estimation. Following ROI selection, $P_{95}$ spatial aggregation and EMA temporal stabilization with α=0.3 were selected during the pilot configuration stage. The $P_{95}$ operator reduces dependence on a single hottest pixel while emphasizing the warmer portion of the ROI, whereas EMA stabilization suppresses short-term frame-to-frame fluctuations with a relatively low mean sample age. Both components represent engineering strategies for temperature extraction and stabilization rather than physiological models of forehead temperature distribution. Application-level evaluation using the medical non-contact infrared forehead thermometer as a practical comparator yielded an overall MAE of 0.514 °C, RMSE of 0.614 °C, and mean bias of −0.187 °C across 90 paired trial-level measurements. Bland–Altman analysis produced limits of agreement from −1.340 °C to 0.966 °C. The relatively small overall mean bias indicates a modest tendency toward underestimation, while the wider limits of agreement indicate substantial variation among individual trials.

Condition-level analysis showed similar mean MAEs for the head pose (0.47 °C), supine (0.48 °C), and baseline (0.49 °C) conditions. Distance variation produced the highest mean MAE (0.58 °C), highest mean RMSE (0.60 °C), and strongest negative bias (−0.58 °C). Every participant showed negative error at 0.5, 1.0, and 1.5 m, with increasing error magnitude across the three distances. The largest individual difference occurred for S10 at 1.5 m, with an error of −1.65 °C. Distance-related behavior was also evident in the direct spatial evaluation. Formal-cohort localization was strongest at 0.5 m, with a mean IoU of 0.752, whereas the mean IoU was 0.695 at both 1.0 and 1.5 m. FIR and NCE did not decrease strictly monotonically across the two farther distances, and the 1.0 m condition showed the greatest NCE variability together with the single spatial failure. The combined spatial and temperature findings therefore indicate reduced robustness at farther camera-to-subject distances relative to 0.5 m, while also showing that distance-related spatial behavior is not strictly monotonic across all localization metrics. Geometric adaptation to apparent nostril scale should consequently not be interpreted as distance-invariant localization or temperature-estimation performance. The head pose condition produced application-level temperature performance comparable to the baseline condition, supporting operation under the moderate natural non-frontal orientations evaluated in this study. Specific yaw, pitch, and roll angles were neither prescribed nor measured; therefore, operational angular limits cannot be established from the present data. Supine acquisition also remained operational, although greater participant-to-participant variability was observed. Direct annotation-based spatial evaluation was not conducted for the head pose or supine conditions, preventing separation of ROI-localization effects from other posture-related sources of temperature variation.

Real-time implementation on the NVIDIA Jetson Orin Nano achieved 24.12±1.43 FPS with a mean system latency of 48.07 ms. Geometric forehead ROI projection required an average of 3.79 ms per frame, corresponding to approximately 7.9% of the measured mean system latency. The relatively small computational contribution of geometric projection supports real-time embedded operation without requiring a separate forehead-specific detection model. Embedded feasibility, however, does not establish long-term monitoring reliability under uncontrolled operating conditions. Noticeable inter-participant variation remained in the temperature-estimation results reported in Table 12. A single fixed geometric configuration was applied across all participants; consequently, differences in craniofacial anatomy, participant positioning, forehead temperature distribution, nasal thermal contrast, and residual ROI localization uncertainty may have contributed to the observed variation. Sequential acquisition and different spatial sampling areas between the handheld thermometer and thermal-camera method may also have contributed. Individual contributions from the potential sources of variation were not isolated in the present evaluation.

Several limitations should therefore be considered when interpreting the results. First, the temperature comparator was a commercially available medical non-contact infrared forehead thermometer rather than a traceably calibrated metrological reference or clinical gold standard. The handheld thermometer also carries measurement uncertainty, and exact spatial correspondence between the two measurement approaches was not possible because of differences in sensing principle, measurement area, and acquisition timing. The reported error metrics and Bland–Altman analysis therefore characterize practical agreement between two forehead temperature measurement approaches under controlled indoor conditions rather than absolute clinical or metrological accuracy. Second, the formal cohort contained only ten healthy adults within a relatively narrow age range and was not stratified by skin tone, ethnicity, BMI, or broader demographic variation. Generalization to children, older adults, clinical populations, or individuals with altered circulation or thermoregulation therefore remains uncertain. Forehead skin temperature can also vary with perfusion, perspiration, ambient temperature, airflow, acclimatization, skin condition, and surrounding thermal sources. Broader environmental variation, substantial occlusion from hair or bedding, masks, strong airflow, large ambient-temperature changes, and prolonged monitoring were not evaluated. Third, direct spatial validation in the formal cohort covered the three frontal camera-to-subject distances but did not include quantitatively defined head orientations or supine posture. Five temporally separated frames per participant and distance were annotated, providing balanced formal-cohort evaluation but not comprehensive characterization of continuous within-subject localization variability. The geometric coefficients also remain fixed population-level approximations rather than participant-specific anatomical parameters. Spatial generalization should therefore be interpreted within the evaluated frontal distance conditions rather than across unrestricted orientations, postures, or populations.

Future work will focus on confidence-aware ROI selection, automatic detection of unreliable projections, adaptive geometric parameter estimation, environmental temperature compensation, and evaluation using larger and more diverse participant cohorts under realistic monitoring conditions. Annotation-based validation will include multiple assessors, quantitatively defined head orientations, upright and supine postures, and a larger number of temporally distributed frames. Evaluation against traceable temperature references and comparison with dedicated deep-learning-based thermal face-localization approaches will also strengthen both spatial and temperature-related validation. Ultimately, the temperature-estimation module will be integrated with the previously developed respiratory rate system [34] and long-lie detection system [36], allowing forehead skin temperature trends to be considered alongside posture, immobility duration, movement, and respiratory status.

## 6. Conclusions

A nostril-anchored geometric ROI projection method was developed for contactless forehead skin temperature estimation using a single low-resolution thermal camera. The framework combines proportional forehead ROI projection, $P_{95}$ spatial aggregation, and EMA temporal stabilization. Direct spatial validation using 150 annotated frames from ten formal participants achieved an overall IoU of 0.714, FIR of 0.870, and NCE of 0.091, with a spatial-failure rate of 0.7%. Localization performance was strongest at 0.5 m and lower at the two farther distances, indicating that geometric adaptation to apparent nostril scale does not provide distance-invariant localization performance. Application-level evaluation across 90 paired measurements produced an overall MAE of 0.514 °C, RMSE of 0.614 °C, and mean bias of −0.187 °C relative to a medical non-contact infrared forehead thermometer used as a practical comparator. The temperature results represent agreement with the comparator rather than absolute clinical or core-temperature accuracy. Real-time implementation achieved 24.12±1.43 FPS on a Jetson Orin Nano, supporting embedded operation. Further validation using larger and more diverse cohorts, defined head orientations and postures, and traceable temperature references remains necessary.

## Figures and Tables

**Table 1:** Comparison of representative ROI localization approaches relevant to thermal forehead temperature estimation.

Method	Anchor/ROI Basis	Resolution Context	Camera Setup	Localization Basis	Embedded Evidence
Manual/semi-automatic ROI analysis [23]	Manually selected forehead/chin ROI	Medium	Single thermal	Operator-defined	Limited
Image-processing-based forehead segmentation [8]	Head ellipse and eye/glasses cues	Low–Medium	Single thermal	Geometric/image-based	Reported
Landmark-based or facial-region localization [24,25,26,27]	Eyes, canthi, or facial landmarks	Medium–High	Single/dual	Landmark-based	Limited
RGB–thermal or multimodal localization [15,26]	RGB landmarks or multimodal face cues	Medium–High	Dual/multimodal	Landmark/ multimodal	Limited
Automatic facial ROI detection [12]	Predefined facial ROIs including forehead	Medium	Single thermal	Learned region labels	Potential
Deep-learning thermal face/facial-region detection [28,29,30,31]	Face or facial regions	Low–Medium	Single thermal	Learned visual features	Reported/ potential
Proposed (this work)	Nostril bounding box and proportional geometric projection	Low	Single thermal	Anthropometry-guided [18]	Evaluated in this study

Note: The reported thermal resolution, sensing configuration, and embedded-deployment evidence vary across the cited studies. This table summarizes characteristics relevant to the present comparison rather than fixed properties of all possible implementations. Because the cited methods were evaluated using different datasets, camera configurations, and assessment criteria, this table provides methodological positioning rather than a direct quantitative ranking. The proposed method was evaluated using a 256 × 192 low-resolution thermal camera.

**Table 2:** Training configuration and validation performance of the YOLOv8n nostril detector.

Training Configuration	
Detector	YOLOv8n
Input image size	640×640 pixels
Epochs	120
Batch size	8
Device	GPU
Data augmentation	Enabled
Augmentation	Horizontal flip, scaling, rotation,
	translation, mosaic, color jitter, and blur
Best Validation Performance	
Selected epoch	74
Precision	0.978
Recall	0.994
mAP@0.5	0.992
mAP@0.5:0.95	0.732

**Table 3:** Geometric parameters used in the orientation-dependent forehead ROI projection.

Parameter	Geometric Function
$k_{1}$	Expansion perpendicular to the projection direction
$k_{2,u}$	ROI extent along the upright projection direction
$k_{3,u}$	Gap between the nostril center and the nearest upright ROI boundary
$k_{2,r}$	ROI extent along the rotated/supine projection direction
$k_{3,r}$	Gap between the nostril center and the nearest rotated/supine ROI boundary

**Table 4:** Thermal camera specifications used in this study.

Parameter	Specification
Camera model	TOPDON TC001
Detector resolution	256×192 pixels
Spectral range	8–14 μm (LWIR)
Frame rate	25 Hz
Thermal sensitivity (NETD)	<40 mK
Nominal accuracy	±2 °C or ±2%
Weight	30 g
Dimensions	71×42×14mm

**Table 5:** Summary of the experimental conditions and evaluation procedures.

Protocol Item	Specification
Common evaluation protocol	
Participants	Ten healthy adults, comprising five females and five males, with a mean age of 36.5±3.8 years. The cohort supported controlled feasibility evaluation without stratification by skin tone, ethnicity, BMI, or broader demographic variation.
Indoor environment	Ambient temperature of approximately 21–23 °C, without strong airflow or a direct heat source near the participant. Heat-emitting objects elsewhere in the scene remained outside the projected forehead ROI and were not sampled during temperature extraction.
Participant preparation	The forehead was exposed to reduce obstruction by hair. No restrictions were imposed regarding facial creams, lotions, cosmetics, or other topical skin products.
Temperature comparator	A medical non-contact infrared forehead thermometer (SPIT003, Guangdong Shipai Technology Co., Ltd., China; device-labelled measurement range: 32.0–42.9 °C; measurement distance: 3–5 cm; measurement time: approximately 1 s) was directed towards the central forehead.
Measurement sequence	The handheld reading was obtained first while the required posture and head orientation were maintained. Thermal acquisition began immediately afterwards without intentional repositioning.
Spatial correspondence	The handheld thermometer was directed towards the central forehead region corresponding as closely as practicable to the projected ROI. Exact spatial correspondence was not assumed because the handheld thermometer produced one temperature reading from the measurement area, whereas the thermal-camera method aggregated multiple temperature pixels within a two-dimensional ROI.
Fixed offset adjustment	A single fixed offset was derived from one initial paired measurement as the difference between the handheld comparator reading and the mean of 30 temporally stabilized thermal estimates. The same offset was retained for all subsequent recordings without recalculation for individual participants or trials.
Warm-up	The first 50 frame-level temperature estimates of each recording were used for initialization and were excluded from subsequent trial-level analysis.
Steady-state criterion	The first 30-sample rolling window with a standard deviation below 0.15 °C defined the start of the steady-state segment. All subsequent temporally stabilized samples were averaged to obtain the trial-level estimate.
Head-orientation assessment	Natural minor movement and non-frontal head orientations were evaluated. Specific yaw, pitch, and roll angles were neither prescribed nor measured.
Acquisition conditions	
Baseline seated	Two 60 s blocks with the participant seated and facing the camera.
Distance variation	Three 60-s blocks acquired at camera-to-subject distances of 0.5, 1.0, and 1.5 m.
Head pose variation	Two 60 s blocks involving minor natural head movement or a non-frontal orientation.
Supine	Two 60 s blocks with the participant lying in a supine posture.

**Table 6:** ROI methods evaluated during the pilot configuration stage.

Method	Implementation
Fixed image-frame crop	A fixed ROI with width 0.15W and height 0.08H, centered horizontally and positioned at 0.15H from the upper image boundary. The crop did not adapt to face position, apparent scale, or camera distance.
MediaPipe Pose-based ROI	A lightweight cross-domain landmark baseline using the left- and right-eye landmarks. The ROI width and height were defined as $0.8d_{\mathrm{eye}}$ and $0.5d_{\mathrm{eye}}$, respectively, and positioned $1.2d_{\mathrm{eye}}$ above the eye center. No thermal-domain fine-tuning was performed.
Proposed nostril-anchored ROI	A forehead ROI with position and dimensions defined proportionally from the tracked nostril bounding box using the geometric formulation described in the preceding subsection.

**Table 7:** Temporal stabilization performance using the selected ROI and spatial aggregation configuration.

Method	Parameter	Raw Variance	Smoothed Variance	Variance Reduction (%)	Mean Sample Age (Frames)
MA	M=5	0.287637	0.254180	11.63	2.0
MA	M=10	0.287637	0.220852	23.22	4.5
MA	M=15	0.287637	0.196752	31.60	7.0
EMA	α=0.3	0.287637	0.236082	17.92	2.3
EMA	α=0.2	0.287637	0.213598	25.74	4.0
EMA	α=0.1	0.287637	0.173258	39.76	9.0

**Table 8:** ROI dimensions and temperature-estimation errors for the upright geometric parameter configurations evaluated under the pilot baseline condition.

$k_{1}$	$k_{2,u}$	$k_{3,u}$	P1 ROI (px)	P2 ROI (px)	MAE (°C)	RMSE (°C)
0.6	1.8	2.5	66.5×44.0	65.5×51.4	0.737	0.839
0.7	2.0	2.8	74.5×48.9	73.6×58.4	0.751	0.848
0.8	2.2	3.0	80.4×53.7	79.3×63.5	0.726	0.817

**Table 9:** Overall direct spatial localization performance across the 120 annotated pilot frames.

Method	Frames	IoU	FIR	NCE	Spatial Failure (%)
Proposed	120	0.603±0.150	0.704±0.161	0.146±0.138	0.8
Fixed crop	120	0.029±0.065	0.062±0.142	2.893±1.742	100.0
MediaPipe Pose	120	0.224±0.170	0.415±0.270	0.354±0.196	69.2

**Table 10:** Distance-dependent performance of the proposed forehead ROI projection and nostril detection.

Distance	Frames	ROI IoU	ROI FIR	ROI NCE	Detection (%)	Confidence	Nostril IoU
0.5 m	40	0.73±0.10	0.84±0.08	0.08±0.04	100	0.83±0.01	0.80±0.07
1.0 m	40	0.54±0.11	0.63±0.12	0.17±0.05	100	0.84±0.03	0.78±0.08
1.5 m	40	0.54±0.15	0.64±0.16	0.19±0.21	100	0.82±0.01	0.75±0.07

**Table 11:** Direct spatial localization performance of the proposed method in the ten-participant formal evaluation cohort.

Distance	Participants	Frames	IoU	FIR	NCE	Spatial Failure (%)
0.5 m	10	50	0.752±0.093	0.905±0.053	0.069±0.036	0.0
1.0 m	10	50	0.695±0.063	0.828±0.082	0.114±0.087	2.0
1.5 m	10	50	0.695±0.048	0.876±0.069	0.088±0.021	0.0
Overall	10	150	0.714±0.035	0.870±0.054	0.091±0.028	0.7

Values for IoU, FIR, and NCE are reported as mean ± standard deviation across the ten participant-level means. Each participant contributed five annotated frames at each distance and 15 frames overall. Spatial failure represents the proportion of individual frames with IoU <0.30.

**Table 12:** Subject-level forehead temperature-estimation performance across the evaluated experimental conditions, with the distance condition separated according to camera-to-subject distance.

	Baseline			Distance 0.5 m			Distance 1.0 m			Distance 1.5 m			Head Pose			Supine		
Subj.	MAE	RMSE	Bias	MAE	RMSE	Bias	MAE	RMSE	Bias	MAE	RMSE	Bias	MAE	RMSE	Bias	MAE	RMSE	Bias
S1	0.01	0.02	+0.01	0.04	0.04	−0.04	0.17	0.17	−0.17	0.55	0.55	−0.55	0.10	0.12	+0.10	0.09	0.11	−0.07
S2	0.53	0.53	−0.53	0.19	0.19	−0.19	0.47	0.47	−0.47	0.65	0.65	−0.65	0.63	0.63	−0.63	0.25	0.26	−0.25
S3	0.35	0.35	−0.35	0.37	0.37	−0.37	0.53	0.53	−0.53	0.63	0.63	−0.63	0.41	0.41	−0.41	0.21	0.22	+0.21
S4	0.33	0.34	+0.33	0.07	0.07	−0.07	0.10	0.10	−0.10	0.15	0.15	−0.15	0.14	0.17	−0.14	0.59	0.59	+0.59
S5	0.46	0.46	−0.46	1.01	1.01	−1.01	1.18	1.18	−1.18	1.23	1.23	−1.23	0.93	0.94	−0.93	0.38	0.38	+0.38
S6	0.52	0.52	+0.52	0.60	0.60	−0.60	0.72	0.72	−0.72	0.74	0.74	−0.74	0.52	0.52	+0.52	1.03	1.03	−0.02
S7	0.22	0.25	+0.22	0.46	0.46	−0.46	0.65	0.65	−0.65	0.70	0.70	−0.70	0.54	0.54	+0.54	0.14	0.14	+0.14
S8	0.67	0.67	+0.67	0.07	0.07	−0.07	0.35	0.35	−0.35	0.51	0.51	−0.51	0.33	0.34	+0.33	1.28	1.28	+1.28
S9	1.00	1.00	−1.00	0.61	0.61	−0.61	0.65	0.65	−0.65	0.72	0.72	−0.72	0.41	0.42	−0.41	0.19	0.20	+0.19
S10	0.82	0.82	+0.82	0.89	0.89	−0.89	0.96	0.96	−0.96	1.65	1.65	−1.65	0.72	0.79	−0.72	0.69	0.73	−0.69

**Table 13:** Condition-level forehead temperature-estimation performance averaged across the ten participants. The reported standard deviations represent variability across participant-level error values.

Condition	MAE ± SD (°C)	RMSE ± SD (°C)	Bias (°C)
Steady baseline	0.49 ± 0.29	0.50 ± 0.29	+0.02
Distance variation	0.58 ± 0.33	0.60 ± 0.33	−0.58
Head pose variation	0.47 ± 0.25	0.49 ± 0.26	−0.17
Supine	0.48 ± 0.41	0.49 ± 0.40	+0.18

**Table 14:** Embedded system performance of the proposed method.

Metric	Value
Frames processed	4788
Recording duration (s)	198.5
Average FPS	24.12±1.43
Mean system latency (ms)	48.07
Median latency (ms)	60.53
P95 latency (ms)	95.98
CPU utilization (%)	36.85
GPU utilization (%)	22.46 mean; 99.50 peak
Memory usage (MB)	1104.8

## Data Availability

The data presented in this study are available on request from the corresponding author.
